# Semen quality changes during infection and recovery phases of mild-to-moderate COVID-19 in reproductive-aged patients: a prospective case series

**DOI:** 10.1186/s12610-022-00175-7

**Published:** 2023-01-19

**Authors:** Nasreldin Mohammed, Mostafa Kamel, Rabea Ahmed Gadelkareem, Mohammed Ali Zarzour, Adel Kurkar, Ahmed Mohammed Abdel-Moniem, Hosny Behnsawy

**Affiliations:** Assiut Urology and Nephrology Hospital, Faculty of Medicine, Assiut University, Elgamaa Street, Assiut, Egypt

**Keywords:** COVID-19, Fertility, Semen analysis, Spermatozoa, Testis, COVID-19, Fertilité, Analyse du Sperme, Spermatozoïdes, Testicule

## Abstract

**Background:**

Despite the documented effects of the coronavirus disease 2019 (COVID-19) on spermatogenesis, the reversibility of these effects is uncertain. We aimed to assess the changes of sperm quality between the infection and recovery phases of COVID-19 in reproductive-aged men. The semen quality of men with mild-to-moderated COVID-19 (defined by the degrees of symptoms and chest involvement on computed tomography) was studied during October, 2020–May, 2021 at our hospital. Two semen samples were analyzed at timings estimated to represent spermatogenic cycles during the infection and recovery phases of COVID-19.

**Results:**

A total of 100 patients were included with mean ± SD (range) age of 24.6 ± 3.3 (21–35) years. During infection, 33% of patients had abnormal semen quality. However, a significant reduction was found in this abnormality from 33 to 11% (*P* < 0.001) after recovery from infection. In a comparison of the two semen analyses, there were significant improvements in the mean values of sperm progressive motility (*P* =0.043) and normal morphology (*P* < 0.001). However, the mean sperm concentration showed a statistically insignificant increase (*P* = 0.844).

**Conclusions:**

In reproductive-aged patients with mild-to-moderate COVID-19, the effects on seminal quality were recoverable, represented by significant improvements in the means of progressive sperm motility and normal morphology between the infection and recovery phases of COVID-19.

**Trial registration:**

ClinicalTrials, NCT04595240.

**Supplementary Information:**

The online version contains supplementary material available at 10.1186/s12610-022-00175-7.

## Résumé

**Contexte:**

Malgré les effets documentés de la maladie à coronavirus 2019 (COVID-19) sur la spermatogenèse, la réversibilité de ces effets reste incertaine. Notre objectif était d’évaluer les changements de la qualité du sperme entre les phases d’infection et de récupération de la COVID-19 chez des hommes en âge de procréer. La qualité du sperme d’hommes atteints d’une forme de COVID-19 de légère à modérée (définie par les degrés de symptômes et l’atteinte thoracique lors de la tomodensitométrie) a été étudiée entre octobre 2020 et mai 2021 dans notre hôpital. Deux échantillons de sperme ont été analysés à des moments estimés représenter les cycles de spermatogénèse pendant les phases d’infection et de rétablissement de la COVID-19.

**Résultats:**

Au total, 100 patients ont été inclus avec un âge moyen ± ET (intervalle) de 24,6 ± 3,3 ans (21-35). Au cours de l’infection, 33% des patients avaient une qualité anormale de sperme. Une réduction significative de cette anomalie de 33 à 11% (*p* < 0,001) a toutefois été observée après la guérison de l’infection. La comparaison des deux analyses de sperme montre des améliorations significatives des valeurs moyennes de la motilité progressive (*p* = 0,043) et de la morphologie normale (p< 0,001) des spermatozoïdes. Cependant, la concentration moyenne de spermatozoïdes a montré une augmentation statistiquement non significative (*p* = 0,844).

**Conclusions:**

Chez les patients en âge de procréer atteints d’une forme de COVID-19 légère à modérée, les effets sur la qualité spermatique étaient récupérables, avec des améliorations significatives des valeurs moyennes de la motilité progressive et de la morphologie normale des spermatozoïdes entre les phases d’infection et de récupération de la COVID-19.

## Background

The severe acute respiratory syndrome coronavirus 2 (SARS-CoV-2) is the organism responsible for the pandemic of the coronavirus diseases 2019 (COVID-19). In the context of this pandemic of COVID-19 and its multi-organ affections, the effects on the male reproductive system have become very important. The research on the effects of COVID-19 on the reproductive system has been directed to the underlying mechanisms of action and pathological and clinical changes, including fertility capabilities and hormonal profiles [1–3]. Focused studying of the effects of COVID-19 on male reproduction is warranted for clarification of the unsettled potential risks of SARS-CoV-2 infection to the male reproductive system [2, 4]. It has been noticed that males of reproductive age represent a significant proportion of the population studied for the clinical characteristics of patients with COVID-19 and the effects on their reproductive system may continue until recovery [5]. Spermatogenesis is the biological process of the male that could primarily be affected by COVID-19. The indicator of the spermatogenic state in patients with COVID-19 is seminal quality. Although SARS-CoV-2 influence on the male reproductive system has been mostly documented, its long-term changes are still unclear [1, 2, 6]. Hence, we conducted the current study to assess the effect of COVID-19 on semen parameters in young adults who tested positive for COVID-19.

### Patients and Methods

A prospective case series was conducted targeting male patients with a confirmed COVID-19 infection who were managed at our hospital during October, 2020–May, 2021. The sample size was calculated using EasyMedStat version 3.17 (www.easymedstat.com). A study power of 80%, margin of error 10%, confidence level of 95%, and probability value 0.05 were considered in the calculation. Considering the percentage of the lost-to-follow-up patients, 100 patients were included and all of them completed this study.

The inclusion criteria were patients aged 20–40 years who had COVID-19 positive reverse transcriptase-polymerase chain reaction (RT-PCR) results on nasopharyngeal swap and categories 2–4 pulmonary involvements of the COVID-19 Reporting and Data System (CO-RAD) in chest computed tomography [7]. The exclusion criteria were patients with a history of previous seminal disorders, testicular surgery, testicular anomalies or diseases such as varicoceles, abnormal secondary sexual characters, severe COVID-19 symptoms (such as respiratory distress warranting oxygen therapy and fever), major comorbidities (such as diabetic, hypertensive and cardiac disorders), or special habits such as smoking and drugs or alcohol addiction. The patients were recruited when they were referred to our hospital for the investigation of COVID-19 by computed tomography and RT-PCR, including both the symptomatic and asymptomatic patients.

**Fig. 1:**
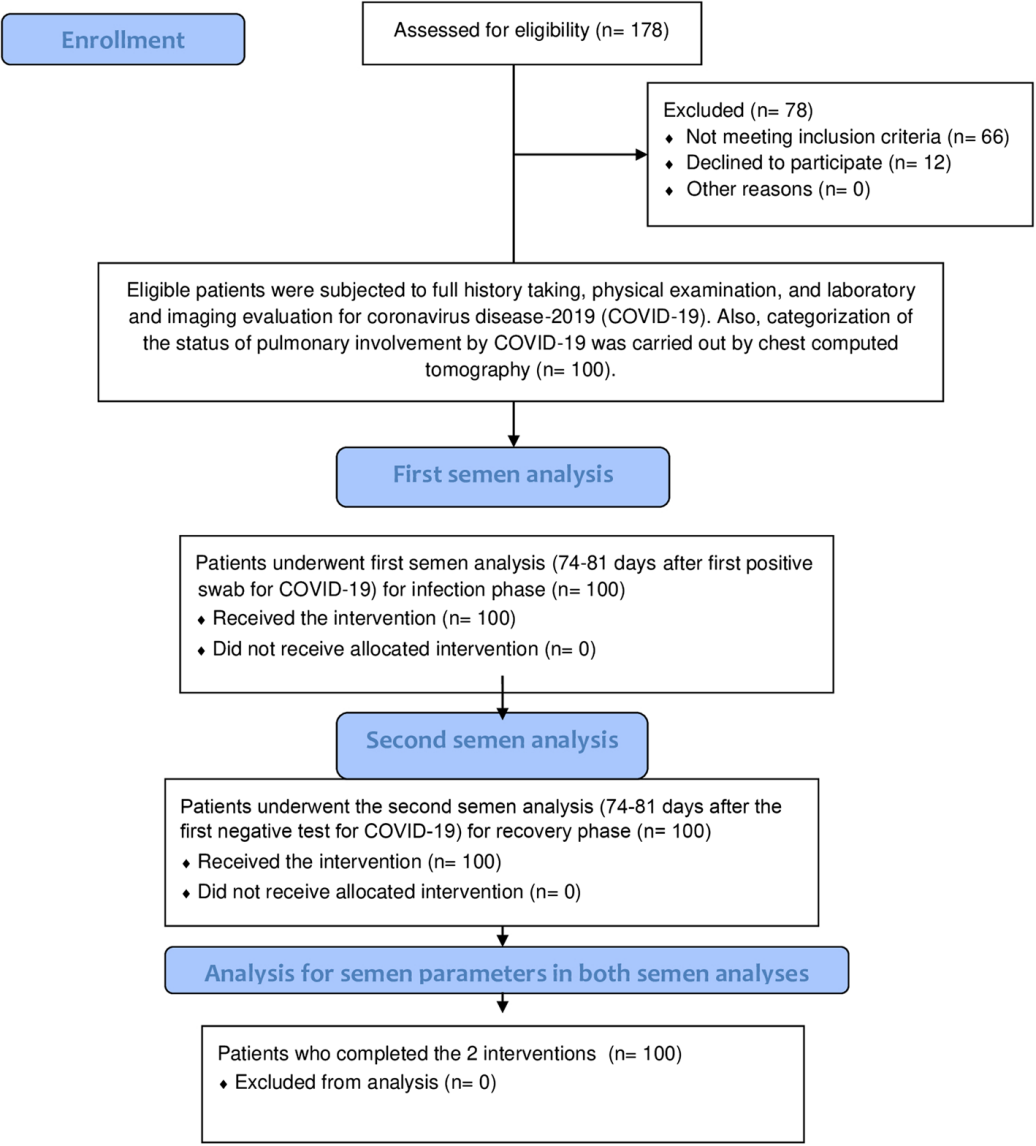
A flowchart of patients with coronavirus diseases-2019 who underwent a clinical evaluation for spermatogenesis via two consecutive semen analyses, representing the infection and recovery phases

All patients were subjected to a full history taking, general examination and laboratory examination including semen analysis. Two samples of semen were analyzed for each participant; the first was 74–81 days after the first positive swab for COVID-19 to represent the state of spermatogenesis during the infection phase; the other also performed 74–81 days after a confirmed negative status of COVID-19 by RT-PCR assay, to represent the semen quality of the recovery phase and for comparison with the first analysis (Fig. 1). The timing ranges for semen specimens were estimated relative to the estimated duration of spermatogenesis of 74 days [8], the duration of confirmed COVID-19 infection, and an abstinence period of 3–7 days.

Semen specimens were obtained by masturbation and semen volume was measured manually, using a micropipette. A validated automated system was used to evaluate the semen parameters; a computer-assisted semen analysis (CASA) machine (Spermolyzer, MiraLap Comp., Helwan, Cairo, Egypt), using MiraLap sperm counting chamber with a depth 20 μm (MiraLap Comp., Helwan, Cairo, Egypt). Validation of the CASA equipment was performed in accordance with the fifth edition of the World Health Organization laboratory manual for the examination and processing of human semen [9]. Agreement in sperm concentration was performed by quality control (QC) samples for internal control and quality assurance (QA) for external control as described in the literature [10]. The lower limit of sperm concentration was defined as 15 million per ml and the lower limit of the percentage of progressive sperm motility was 32%. The normal form of spermatozoa was assessed according to Kruger’s strict criteria [11], considering the lower limit of the percentage of normal spermatozoa 4%. The assessment of leucocytes in semen was performed by the peroxidase activity assay, defining the upper limit of the normal count of the peroxidase-positive leucocytes as 1 million per ml [9].

The primary outcome of this study was the change in semen quality in two consecutive cycles of spermatogenesis represented by the two semen analyses performed at least 74 days after the dates of confirmed infection and resolution of COVID-19. Abnormal semen quality was defined as the presence of at least one abnormal parameter value per semen analysis.

### Statistical analysis

The Statistical Package for the Social Science (SPSS, IBM, Armonk, New York, USA), version 20, was used in analysis of data. The quantitative data were presented as mean ± standard deviation (range), while the qualitative data were presented as frequency and percentage. The total semen quality was compared by Chi-squared test. However, the pH and volume of semen and progressive sperm motility were compared by paired Student-t test. The other parameters, including sperm concentration and morphology, red blood cells and leucocytes were compared by Wilcoxon test. The level of confidence was adjusted at 95% and *P* value was considered significant when it was < 0.05.

## Results

This study included 100 patients with category 2 to 4 CO-RAD pulmonary involvement COVID-19 on chest computed tomography. The relevant demographic and clinical characteristics are demonstrated (Table 1).

**Table 1:** Demographic and clinical characteristics of COVID-19 patients examined for semen quality (*n*=100)

Characteristics	Value Mean ± SD (Range) or Frequency (%)
Age (y)	24.6 ± 3.3 (21–35)
Height (m)	1.7 ± 0.1 (1.5–1.8)
Weight (kg)	75.4 ± 10.4 (55–99)
Body mass index	26 ± 3.7 (18.6–37.1)
Marital status	
Single	38 (38%)
Married	53 (53%)
Divorced/Widow	9 (9%)
Duration of confirmed infection (days)	17 ± 5.5 (7–33)
Duration between disease confirmation and first semen analysis (days)	77.2 ± 1.9 (75–81)
Duration between first and second semen analyses (days)	92.8 ± 5.9 (81–110)
CO-RAD category by chest computed tomography	
CO-RAD 2	25 (25%)
CO-RAD 3	54 (54%)
CO-RAD 4	21 (21%)

*CO-RAD* COVID-19 Reporting and Data System, *COVID-19* Coronavirus disease 2019

Representing the infection phase, semen parameters in the first analysis showed 33% of patients had abnormal quality. The second semen analysis during the recovery phase, however, showed a significant reduction in the percentage of abnormal semen quality, returning to 11% (*P* < 0.001). The comparison of semen parameters in these two analyses showed significant improvement in the mean values of progressive sperm motility (*P* =0.043) and normal morphology (*P* < 0.001). The mean sperm concentration showed an improvement in the second analysis, but it was statistically insignificant (*P* =0.844) (Table 2).

**Table 2:** Comparison between semen parameters of the first (representing the infection phase) and second (representing the recovery phase) semen analyses^a^

Semen parameters	First analysis	Second analysis	*P*-value
	Mean ± SD (Range) or Frequency (Percentage)		
pH	7.6 ± 0.3 (7–8)	7.6 ± 0.3 (7–8)	0.581
Volume (mL)	3.27 ± 0.75 (2–4.6)	3.25 ± 0.76 (2–4.6)	0.813
Sperm concentration (million/mL)	96.5 ± 35.9 (12–154)	104.7 ± 33.8 (15–154)	0.844
Percentage of progressive sperm motility	44.5 ± 6.7 (34–58)	46.5 ± 7.1 (34–58)	0.043
Morphology (% of normal forms)	23.4 ± 17.6 (1–55)	30.6 ± 13 (2–60)	<0.001
Red blood cells (10^6^/ml)	1.07 ± 0.78 (0–2)	1.06 ± 0.76 (0–2)	0.877
Leucocytes (10^6^/ml)	1.2 ± 1 (0–6)	1.1 ± 0.8 (0–2)	0.539
Total semen quality^b^			
Normal	67 (67%)	89 (89%)	<0.001
Abnormal	33 (33%)	11 (11%)	

^a^The pH and volume of semen and the progressive sperm motility were compared, using the paired Student-t test. The other parameters, including sperm concentration and morphology, red blood cells and leucocytes were compared by Wilcoxon test. In addition, the total semen quality was compared, using the Chi-squared test.

^b^Total semen quality: This expression considered the quality of semen as a whole (normal or abnormal) relative to the presence or absence of the abnormality, regardless the number of the abnormal parameters.

*Abbreviations*: *pH* the potential hydrogen describing the acidity or basicity of semen, *SD* Standard deviation

## Discussion

The cell surface receptors of angiotensin-converting enzyme 2 (ACE2) and transmembrane serine protease 2 have been proven to be the binding components of SARS-CoV-2 entry to the host cells and integration of its contents for replication [12]. Therefore, the higher sex-based susceptibility to catch COVID-19 disease among males could be attributed to the higher levels of ACE2 in males than females. Males may also have lower capabilities of viral load clearance than females [2], and hence the testes should be regarded as high-risk organs and potential targets for COVID-19 in males at the reproductive age [5, 13]. Many authors have shown concern that the majority of COVID-19 patients fall within the reproductive age category, ranging from 15–49 years [5, 6]. This risk was a strong motivator to conduct the current study on patients in this age category. We used CO-RAD to define the mild-to-moderate pulmonary involvement as the most common tool of diagnosis and as an indicator of the disease burden. Moreover, exclusion of patients with hypoxia and fever eliminated their proposed confounding effects on spermatogenesis with the COVID-19 effect [1].

On the other hand, the current study employed strict patient selection criteria to avoid the confounding factors. These criteria provided a sample of patients with demographic and clinical characteristics that may not be representative to their corresponding groups in the general populations. Among these characteristics, the body mass index (BMI) is slightly higher than that of the average population of the young adults. This can be attributed to that the potential associations between the high BMI and COVID-19 infections, regarding the severity and mortality of COVID-19 [14]. The means of normal sperm morphology in the two seminal analyses were higher than 20% which were significantly higher than the known means in the fertile males [9]. Similarly, this finding can be attributed to the strict exclusion criteria which resulted in exclusion of patients with comorbidities that may influence the seminal quality, including varicoceles, previous surgeries, chronic systemic diseases, and any other testicular diseases.

The mechanisms of action by COVID-19 so far considered include multiple pathways of pathophysiological alterations. Direct invasion and damage of the testis has recently been proven [2, 12]. SARS-CoV-2 disturbs the immunological characteristics and provokes high systemic levels of the inflammatory mediators, which include the pro-inflammatory cytokines, defined as a ‘cytokine storm’ effect, and disturbed seminal antioxidant defense mechanisms [4, 6]. The constitutional stressors of COVID-19 such as fever, hypoxia and medications, may also play a role in the effects on spermatogenesis in those patients [2, 5]. Therefore, we excluded patients with severe clinical symptoms, to avoid their confounding effects on semen quality.

Despite this evidence of influences on the male reproductive system by various mechanisms, the presence of SARS-CoV-2 in the testis and semen of COVID-19 patients remains a matter of debate [13, 15]. Most reports in the literature have reported that SARS-CoV-2 is undetectable in the semen samples of COVID-19 patients [16–18]. On the other hand, patients’ semen parameters have been found to be significantly affected by SARS-CoV-2 infection, resulting in oligoasthenoteratozoospermia or one of its components as spermatogenic outcomes [6, 16, 19]. On the clinical and academic levels, this has raised a major concern whether COVID-19 should clinically be flagged as a cause of male subfertility with uncertain duration of recoverability [6, 19, 20]. Our study considered this clinical concern and compared semen parameter values of two consecutive spermatogenic cycles, representing the infection and recovery phases of COVID-19 in a duration of 5–6 months.

While many studies have reported semen quality changes during COVID-19 infection, studying the long-term reversibility is warranted [1, 6, 15, 16, 19]. In one study of semen quality, COVID-19 patients had significantly lower values of semen volume, progressive sperm motility, normal sperm morphology, sperm concentration, and number of spermatozoa relative to the control group. Their results showed enhanced numbers of spermatozoa and percentages of progressive motility in semen analyses performed through the following 60 days, relative to the baseline value among the COVID-19 patients. Although they observed a form of improvement of semen quality towards normal, full recoverability of these parameters seemed uncertain within 60 days [6]. The current results come in parallel with these findings, where the improvement was significantly better with longer duration after COVID-19 resolution. Li et al. [1] reported that 39.1% of hospitalized patients with COVID-19 had oligozoospermia, attributing impairment of spermatogenesis to an elevated immune response and autoimmune orchitis [1]. Other researchers have found significant changes in sperm morphology during the infection phase of the disease and attributed them to the acute stress of COVID-19 [16, 19]. Similarly, the current results revealed that the changes in sperm morphology were the most prominent abnormality in sperm characteristics during the infection phase with significant improvement after recovery from the disease.

The pathophysiological mechanisms of testicular damage by COVID-19, including the relations between the receptors and frequent viral mutations, represent a principal point in the relevant literature. The effects of COVID-19 on the hypothalamic-pituitary-gonadal axis have been addressed by the ongoing studying. This is in concordance to the research efforts required for testicular protection against COVID-19 by developing standardized treatment strategies [2, 21]. Regarding the effects on fertility outcomes, the clinical aspect of testicular effects of COVID-19 is primarily directed towards its potential effects on semen quality. However, the long-term effect is not yet fully understood [2–4]. Hence, the current study may be useful in providing a step forward in studying the temporal effects of COVID-19 on spermatogenesis.

The limitations of the current study include the non-testing of the hormonal profiles of those patients, and the lack of baseline semen quality, which may have detected spermatogenic defects before disease onset. Although the exclusion of patients with severe COVID-19 was to avoid the effect of the constitutional effects of the disease on spermatogenesis, it is a form of selection bias. However, this should not be the important, because the current study and the results were strictly relevant to the patients with mild-to-moderate COVID-19.

## Conclusions

Semen quality was abnormal in 33% of reproductive-aged patients with category 2-4 CO-RAD pulmonary involvement during the infection phase of COVID-19. There was a significant reduction of the percentages of men with abnormal semen quality after recovery from infection, with significant improvements of mean semen progressive motility and normal morphology during the estimated duration of two consecutive spermatogenesis cycles. Hence, the impairment of spermatogenesis due to COVID-19 seems to be reversible.

## Supplementary Information

**Additional file 1.**

**Additional file 2.**

**Additional file 3.**

## Data Availability

The data used and analyzed during the current study are available from the corresponding author on reasonable request.

## Abbreviations

ACE2: Angiotensin-converting enzyme 2
BMI: Body mass index
CO-RAD: COVID-19 Reporting and Data System
COVID-19: Coronavirus diseases 2019
RT-PCR: Reverse transcriptase-polymerase chain reaction assay
SARS-CoV-2: Severe acute respiratory syndrome coronavirus 2
