# Supplementary material for: Semen quality changes during infection and recovery phases of mild-to-moderate COVID-19 in reproductive-aged patients: a prospective case series
Source: Basic Clin Androl. 2023 Jan 19;33:2. doi: 10.1186/s12610-022-00175-7 (PMC9848703; doi:10.1186/s12610-022-00175-7)
Supplement: Supplementary file 1 — Additional file 1. [file 12610_2022_175_MOESM1_ESM.docx]

**Semen Quality Changes during Infection and Recovery Phases of Mild-to-Moderate COVID-19 in Reproductive-Aged Patients: A Prospective Case Series**

Authors:

Nasreldin Mohammed^1^, Mostafa Kamel^1^, Rabea Ahmed Gadelkareem^1,*^, Mohammed Ali Zarzour^1^, Adel Kurkar^1^, Ahmed Mohammed Abdel-Moniem^1^, Hosny Behnsawy^1^

Affiliation:

^1^ Assiut Urology and Nephrology Hospital, Faculty of Medicine, Assiut University, Egypt

*: **Corresponding author:** Rabea Ahmed Gadelkareem

Postal address: Elgamaa Street, Assiut University, Assiut, Egypt.

Postal code: 71515; Tel.: +20 0882080435; Fax: +20 0882080236

E-mail: dr.rabeagad@yahoo.com; rabeagad@aun.edu.eg

ORCID: 0000-0003-4403-2859

**Abstract**

**Background**: Despite the documented effects of the coronavirus disease 2019 (COVID-19) on spermatogenesis, the reversibility of these effects is uncertain. We aimed to assess the changes of sperm quality between the infection and recovery phases of COVID-19 in reproductive-aged men. The semen quality of men with mild-to-moderated COVID-19 (defined by the degrees of symptoms and chest involvement on computed tomography) was studied during October, 2020–May, 2021 at our hospital. Two semen samples were analyzed at timings estimated to represent spermatogenic cycles during the infection and recovery phases of COVID-19.

**Results:** A total of 100 patients were included with mean ± SD (range) age of 24.6 ± 3.3 (21–35) years. During infection, 33% of patients had abnormal semen quality. However, a significant reduction was found in this abnormality from 33 to 11% (P < 0.001) after recovery from infection. In a comparison of the two semen analyses, there were significant improvements in the mean values of sperm progressive motility (P =0.043) and normal morphology (P < 0.001). However, the mean sperm concentration showed a statistically insignificant increase (P = 0.844).

**Conclusions:** In reproductive-aged patients with mild-to-moderate COVID-19, the effects on seminal quality were recoverable, represented by significant improvements in the means of progressive sperm motility and normal morphology between the infection and recovery phases of COVID-19.

**Trial registration:** ClinicalTrials, NCT04595240. https://clinicaltrials.gov/ct2/history/NCT04595240?V_1=View#StudyPageTop

**Keywords:** COVID-19; Fertility; Semen analysis; Spermatozoa; Testis

**Background**

The severe acute respiratory syndrome coronavirus 2 (SARS-CoV-2) is the organism responsible for the pandemic of the coronavirus diseases 2019 (COVID-19). In the context of this pandemic of COVID-19 and its multi-organ affections, the effects on the male reproductive system have become very important. The research on the effects of COVID-19 on the reproductive system has been directed to the underlying mechanisms of action and pathological and clinical changes, including fertility capabilities and hormonal profiles [1-3]. Focused studying of the effects of COVID-19 on male reproduction is warranted for clarification of the unsettled potential risks of SARS-CoV-2 infection to the male reproductive system [2, 4]. It has been noticed that males of reproductive age represent a significant proportion of the population studied for the clinical characteristics of patients with COVID-19 and the effects on their reproductive system may continue until recovery [5]. Spermatogenesis is the biological process of the male that could primarily be affected by COVID-19. The indicator of the spermatogenic state in patients with COVID-19 is seminal quality. Although SARS-CoV-2 influence on the male reproductive system has been mostly documented, its long-term changes are still unclear [1, 2, 6]. Hence, we conducted the current study to assess the effect of COVID-19 on semen parameters in young adults who tested positive for COVID-19.

**Patients and Methods**

A prospective case series was conducted targeting male patients with a confirmed COVID-19 infection who were managed at our hospital during October, 2020–May, 2021. The sample size was calculated using EasyMedStat version 3.17 (www.easymedstat.com). A study power of 80%, margin of error 10%, confidence level of 95%, and probability value 0.05 were considered in the calculation. Considering the percentage of the lost-to-follow-up patients, 100 patients were included and all of them completed this study.

The inclusion criteria were patients aged 20‒40 years who had COVID-19 positive reverse transcriptase-polymerase chain reaction (RT-PCR) results on nasopharyngeal swap and categories 2–4 pulmonary involvements of the COVID-19 Reporting and Data System (CO-RAD) in chest computed tomography [7]. The exclusion criteria were patients with a history of previous seminal disorders, testicular surgery, testicular anomalies or diseases such as varicoceles, abnormal secondary sexual characters, severe COVID-19 symptoms (such as respiratory distress warranting oxygen therapy and fever), major comorbidities (such as diabetic, hypertensive and cardiac disorders), or special habits such as smoking and drugs or alcohol addiction. The patients were recruited when they were referred to our hospital for the investigation of COVID-19 by computed tomography and RT-PCR, including both the symptomatic and asymptomatic patients.

All patients were subjected to a full history taking, general examination and laboratory examination including semen analysis. Two samples of semen were analyzed for each participant; the first was 74–81 days after the first positive swab for COVID-19 to represent the state of spermatogenesis during the infection phase; the other also performed 74–81 days after a confirmed negative status of COVID-19 by RT-PCR assay, to represent the semen quality of the recovery phase and for comparison with the first analysis (Figure 1). The timing ranges for semen specimens were estimated relative to the estimated duration of spermatogenesis of 74 days [8], the duration of confirmed COVID-19 infection, and an abstinence period of 3–7 days.

Semen specimens were obtained by masturbation and semen volume was measured manually, using a micropipette. A validated automated system was used to evaluate the semen parameters; a computer-assisted semen analysis (CASA) machine (Spermolyzer, MiraLap Comp., Helwan, Cairo, Egypt), using MiraLap sperm counting chamber with a depth 20 µm (MiraLap Comp., Helwan, Cairo, Egypt). Validation of the CASA equipment was performed in accordance with the fifth edition of the World Health Organization laboratory manual for the examination and processing of human semen [9]. Agreement in sperm concentration was performed by quality control (QC) samples for internal control and quality assurance (QA) for external control as described in the literature [10]. The lower limit of sperm concentration was defined as 15 million per ml and the lower limit of the percentage of progressive sperm motility was 32%. The normal form of spermatozoa was assessed according to Kruger’s strict criteria [11], considering the upper limit of the percentage of normal spermatozoa 4%. The assessment of leucocytes in semen was performed by the peroxidase activity assay, defining the upper limit of the normal count of the peroxidase-positive leucocytes as 1 million per ml [9].

The primary outcome of this study was the change in semen quality in two consecutive cycles of spermatogenesis represented by the two semen analyses performed at least 74 days after the dates of confirmed infection and resolution of COVID-19. Abnormal semen quality was defined as the presence of at least one abnormal parameter value per semen analysis.

Statistical analysis: The Statistical Package for the Social Science (SPSS, IBM, Armonk, New York, USA), version 20, was used in analysis of data. The quantitative data were presented as mean ± standard deviation (range), while the qualitative data were presented as frequency and percentage. The total semen quality was compared by Chi-squared test. However, the pH and volume of semen and progressive sperm motility were compared by paired Student-t test. The other parameters, including sperm concentration and morphology, red blood cells and leucocytes were compared by Wilcoxon test. The level of confidence was adjusted at 95% and P value was considered significant when it was < 0.05.

**Results**

This study included 100 patients with category 2 to 4 CO-RAD pulmonary involvement COVID-19 on chest computed tomography. The relevant demographic and clinical characteristics are demonstrated (Table 1).

Representing the infection phase, semen parameters in the first analysis showed 33% of patients had abnormal quality. The second semen analysis during the recovery phase, however, showed a significant reduction in the percentage of abnormal semen quality, returning to 11% (P < 0.001). The comparison of semen parameters in these two analyses showed significant improvement in the mean values of progressive sperm motility (P =0.043) and normal morphology (P < 0.001). The mean sperm concentration showed an improvement in the second analysis, but it was statistically insignificant (P =0.844) (Table 2).

**Discussion**

The cell surface receptors of angiotensin-converting enzyme 2 (ACE2) and transmembrane serine protease 2 have been proven to be the binding components of SARS-CoV-2 entry to the host cells and integration of its contents for replication [12]. Therefore, the higher sex-based susceptibility to catch COVID-19 disease among males could be attributed to the higher levels of ACE2 in males than females. Males may also have lower capabilities of viral load clearance than females [2], and hence the testes should be regarded as high-risk organs and potential targets for COVID-19 in males at the reproductive age [5, 13]. Many authors have shown concern that the majority of COVID-19 patients fall within the reproductive age category, ranging from 15‒49 years [5, 6]. This risk was a strong motivator to conduct the current study on patients in this age category. We used CO-RAD to define the mild-to-moderate pulmonary involvement as the most common tool of diagnosis and as an indicator of the disease burden. Moreover, exclusion of patients with hypoxia and fever eliminated their proposed confounding effects on spermatogenesis with the COVID-19 effect [1].

On the other hand, the current study employed strict patient selection criteria to avoid the confounding factors. These criteria provided a sample of patients with demographic and clinical characteristics that may not be representative to their corresponding groups in the general populations. Among these characteristics, the body mass index (BMI) is slightly higher than that of the average population of the young adults. This can be attributed to that the potential associations between the high BMI and COVID-19 infections, regarding the severity and mortality of COVID-19 [14]. The means of normal sperm morphology in the two seminal analyses were higher than 20% which were significantly higher than the known means in the fertile males [9]. Similarly, this finding can be attributed to the strict exclusion criteria which resulted in exclusion of patients with comorbidities that may influence the seminal quality, including varicoceles, previous surgeries, chronic systemic diseases, and any other testicular diseases.

The mechanisms of action by COVID-19 so far considered include multiple pathways of pathophysiological alterations. Direct invasion and damage of the testis has recently been proven [2, 12]. SARS-CoV-2 disturbs the immunological characteristics and provokes high systemic levels of the inflammatory mediators, which include the pro-inflammatory cytokines, defined as a ‘cytokine storm’ effect, and disturbed seminal antioxidant defense mechanisms [4, 6]. The constitutional stressors of COVID-19 such as fever, hypoxia and medications, may also play a role in the effects on spermatogenesis in those patients [2, 5]. Therefore, we excluded patients with severe clinical symptoms, to avoid their confounding effects on semen quality.

Despite this evidence of influences on the male reproductive system by various mechanisms, the presence of SARS-CoV-2 in the testis and semen of COVID-19 patients remains a matter of debate [13, 15]. Most reports in the literature have reported that SARS-CoV-2 is undetectable in the semen samples of COVID-19 patients [16-18]. On the other hand, patients’ semen parameters have been found to be significantly affected by SARS-CoV-2 infection, resulting in oligoasthenoteratozoospermia or one of its components as spermatogenic outcomes [6, 16, 19]. On the clinical and academic levels, this has raised a major concern whether COVID-19 should clinically be flagged as a cause of male subfertility with uncertain duration of recoverability [6, 19, 20]. Our study considered this clinical concern and compared semen parameter values of two consecutive spermatogenic cycles, representing the infection and recovery phases of COVID-19 in a duration of 5–6 months.

While many studies have reported semen quality changes during COVID-19 infection, studying the long-term reversibility is warranted [1, 6, 15, 16, 19]. In one study of semen quality, COVID-19 patients had significantly lower values of semen volume, progressive sperm motility, normal sperm morphology, sperm concentration, and number of spermatozoa relative to the control group. Their results showed enhanced numbers of spermatozoa and percentages of progressive motility in semen analyses performed through the following 60 days, relative to the baseline value among the COVID-19 patients. Although they observed a form of improvement of semen quality towards normal, full recoverability of these parameters seemed uncertain within 60 days [6]. The current results come in parallel with these findings, where the improvement was significantly better with longer duration after COVID-19 resolution. Li et al. [1] reported that 39.1% of hospitalized patients with COVID-19 had oligozoospermia, attributing impairment of spermatogenesis to an elevated immune response and autoimmune orchitis [1]. Other researchers have found significant changes in sperm morphology during the infection phase of the disease and attributed them to the acute stress of COVID-19 [16, 19]. Similarly, the current results revealed that the changes in sperm morphology were the most prominent abnormality in sperm characteristics during the infection phase with significant improvement after recovery from the disease.

The pathophysiological mechanisms of testicular damage by COVID-19, including the relations between the receptors and frequent viral mutations, represent a principal point in the relevant literature. The effects of COVID-19 on the hypothalamic-pituitary-gonadal axis have been addressed by the ongoing studying. This is in concordance to the research efforts required for testicular protection against COVID-19 by developing standardized treatment strategies [2, 21]. Regarding the effects on fertility outcomes, the clinical aspect of testicular effects of COVID-19 is primarily directed towards its potential effects on semen quality. However, the long-term effect is not yet fully understood [2-4]. Hence, the current study may be useful in providing a step forward in studying the temporal effects of COVID-19 on spermatogenesis.

The limitations of the current study include the non-testing of the hormonal profiles of those patients, and the lack of baseline semen quality, which may have detected spermatogenic defects before disease onset. Although the exclusion of patients with severe COVID-19 was to avoid the effect of the constitutional effects of the disease on spermatogenesis, it is a form of selection bias. However, this should not be the important, because the current study and the results were strictly relevant to the patients with mild-to-moderate COVID-19.

**Conclusions**

Semen quality was abnormal in 33% of reproductive-aged patients with category 2-4 CO-RAD pulmonary involvement during the infection phase of COVID-19. There was a significant reduction of the percentages of men with abnormal semen quality after recovery from infection, with significant improvements of mean semen progressive motility and normal morphology during the estimated duration of two consecutive spermatogenesis cycles. Hence, the impairment of spermatogenesis due to COVID-19 seems to be reversible.

**List of abbreviations**

1. ACE2: Angiotensin-converting enzyme 2
2. BMI: Body mass index
3. CO-RAD: COVID-19 Reporting and Data System
4. COVID-19: Coronavirus diseases 2019.
5. RT-PCR: Reverse transcriptase-polymerase chain reaction assay
6. SARS-CoV-2: Severe acute respiratory syndrome coronavirus 2

**Declarations:**

Ethical approval: All procedures performed in studies involving human participants were in accordance with the standards of the ethical committee of the Faculty of Medicine, Assiut University (the institutional review board approval number is 17300689/2021 and ClinicalTrials number is NCT04595240) and with the 1964 Helsinki declaration and its later amendments or comparable ethical standards.

Funding: None.

Disclosure of potential conflicts of interest: The authors declare that they have no competing interests.

Availability of data: The data used and analyzed during the current study are available from the corresponding author on reasonable request.

Informed consent: Informed consent was obtained from all individual participants included in the study.

Aauthors’ Contributions: NM contributed in data collection, writing, revision, and approval. MK contributed in data collection, statistical analysis, writing, and approval. RAG contributed in concept design, data collection, statistical analysis, writing, and approval. MAZ contributed in data collection, statistical analysis, writing, and approval. AK contributed in concept design, writing, statistical analysis, revision, and approval. AMA contributed in concept design, writing, revision, and approval. HB contributed in concept design, writing, revision, and approval. All authors read and approved the final manuscript.

Acknowledgements: Not applicable.

**References**

1. Li H, Xiao X, Zhang J, Zafar MI, Wu C, Long Y, et al. Impaired spermatogenesis in COVID-19 patients. EClinicalMedicine 2020;28:100604. doi: 10.1016/j.eclinm.2020.100604
2. Li X, Chen Z, Geng J, Mei Q, Li H, Mao C, Han M. COVID-19 and Male Reproduction: A Thorny Problem. Am J Mens Health 2022;16:15579883221074816. doi: 10.1177/15579883221074816
3. Banihani SA. Human semen quality as affected by SARS-CoV-2 infection: An up-to-date review. Andrologia 2022;54:e14295. doi: 10.1111/and.14295
4. Sengupta P, Dutta S, Roychoudhury S, D'Souza UJA, Govindasamy K, Kolesarova A. COVID-19, Oxidative Stress and Male Reproduction: Possible Role of Antioxidants. Antioxidants (Basel) 2022;11:548. doi: 10.3390/antiox11030548
5. Guan W, Guan W, Ni Z, Hu Y, Liang W, Ou C, et al. Clinical Characteristics of Coronavirus Disease 2019 in China. N Engl J Med. 2020;382:1708-20. 10.1056/NEJMoa2002032
6. Hajizadeh MB and Tartibian B. COVID-19 and male reproductive function: a prospective, longitudinal cohort study. Reproduction 2021;161:319-31. doi: 10.1530/REP-20-0382
7. Prokop M, van Everdingen W, van Rees Vellinga T, Quarles van Ufford H, Stöger L, Beenen L, et al. COVID-19 Standardized Reporting Working Group of the Dutch Radiological Society. CO-RADS: A Categorical CT Assessment Scheme for Patients Suspected of Having COVID-19-Definition and Evaluation. Radiology 2020;296:E97-104. doi: 10.1148/radiol.2020201473
8. Griswold MD. Spermatogenesis: The Commitment to Meiosis. Physiol Rev. 2016;96:1-17. doi: 10.1152/physrev.00013.2015
9. World Health Organization. WHO laboratory manual for the examination and processing of human semen, 5th ed. World Health Organization. 2010. https://apps.who.int/iris/handle/10665/44261. Accessed 28 Nov 2022.
10. Agarwal A, Sharma R, Gupta S, Finelli R, Parekh N, Selvam MKP, et al. Standardized Laboratory Procedures, Quality Control and Quality Assurance Are Key Requirements for Accurate Semen Analysis in the Evaluation of Infertile Male. World J Mens Health. 2022;40:52-65. doi: 10.5534/wjmh.210022
11. Kruger TF, DuToit TC, Franken DR, Acosta AA, Oehninger SC, Menkveld R, et al. A new computerized method of reading sperm morphology (strict criteria) is as efficient as technician reading. Fertil Steril. 1993;59:202-9. doi: 10.1016/s0015-0282(16)55640-4
12. Hoffmann M, Kleine-Weber H, Schroeder S, Krüger N, Herrler T, Erichsen S, et al. SARS-CoV-2 Cell Entry Depends on ACE2 and TMPRSS2 and Is Blocked by a Clinically Proven Protease Inhibitor. Cell 2020;181:271-80. doi: 10.1016/j.cell.2020.02.052
13. Ma L, Xie W, Li D, Shi L, Ye G, Mao Y, et al. Evaluation of sex-related hormones and semen characteristics in reproductive-aged male COVID-19 patients. J Med Virol. 2021;93:456-62. doi: 10.1002/jmv.26259
14. Yates T, Summerfield A, Razieh C, Banerjee A, Chudasama Y, Davies MJ, et al. A population-based cohort study of obesity, ethnicity and COVID-19 mortality in 12.6 million adults in England. Nat Commun. 2022;13:624. doi: 10.1038/s41467-022-28248-1
15. Holtmann N, Edimiris P, Andree M, Doehmen C, Baston-Buest D, Adams O, et al. Assessment of SARS-CoV-2 in human semen-a cohort study. Fertil Steril. 2020;114:233-8. doi: 10.1016/j.fertnstert.2020.05.028
16. Gacci M, Coppi M, Baldi E, Sebastianelli A, Zaccaro C, Morselli S, et al. Semen impairment and occurrence of SARS-CoV-2 virus in semen after recovery from COVID-19. Hum Reprod. 2021;36:1520-9. doi: 10.1093/humrep/deab026
17. Kayaaslan B, Korukluoglu G, Hasanoglu I, Kalem AK, Eser F, Akinci E, et al. Investigation of SARS-CoV-2 in Semen of Patients in the Acute Stage of COVID-19 Infection. Urol Int. 2020;104:678-83. doi: 10.1159/000510531
18. Pan F, Xiao X, Guo J, Song Y, Li H, Patel DP, et al. No evidence of severe acute respiratory syndrome-coronavirus 2 in semen of males recovering from coronavirus disease 2019. Fertil Steril. 2020;113:1135-9. doi: 10.1016/j.fertnstert.2020.04.024
19. Temiz MZ, Dincer MM, Hacibey I, Yazar RO, Celik C, Kucuk SH, et al. Investigation of SARS-CoV-2 in semen samples and the effects of COVID-19 on male sexual health by using semen analysis and serum male hormone profile: A cross-sectional, pilot study. Andrologia 2021;53:e13912. doi: 10.1111/and.13912
20. Patel DP, Punjani N, Guo J, Alukal JP, Li PS, Hotaling JM. The impact of SARS-CoV-2 and COVID-19 on male reproduction and men’s health. Fertil Steril. 2021;115:813–23. doi: 10.1016/j. fertnstert.2020.12.033
21. Selvaraj K, Ravichandran S, Krishnan S, Radhakrishnan RK, Manickam N, Kandasamy M. Testicular Atrophy and Hypothalamic Pathology in COVID-19: Possibility of the Incidence of Male Infertility and HPG Axis Abnormalities. Reprod Sci. 2021;28:2735-42. doi: 10.1007/s43032-020-00441-x

Figure legend

Figure 1: A flowchart of patients with coronavirus diseases-2019 who underwent a clinical evaluation for spermatogenesis via two consecutive semen analyses, representing the infection and recovery phases
