# Supplementary material for: Semen quality changes during infection and recovery phases of mild-to-moderate COVID-19 in reproductive-aged patients: a prospective case series
Source: Basic Clin Androl. 2023 Jan 19;33:2. doi: 10.1186/s12610-022-00175-7 (PMC9848703; doi:10.1186/s12610-022-00175-7)
Supplement: Supplementary file 2 — Additional file 2. [file 12610_2022_175_MOESM2_ESM.docx]

Table 1: Demographic and clinical characteristics of COVID-19 patients examined for semen quality (n=100).

| Characteristics | Value  Mean ± SD (Range) or Frequency (%) |
| --- | --- |
| Age (y) | 24.6 ± 3.3 (21–35) |
| Height (m) | 1.7 ± 0.1 (1.5–1.8) |
| Weight (kg) | 75.4 ± 10.4 (55–99) |
| Body mass index | 26 ± 3.7 (18.6–37.1) |
| Marital status |  |
| Single | 38 (38%) |
| Married | 53 (53%) |
| Divorced/Widow | 9 (9%) |
| Duration of confirmed infection (days) | 17 ± 5.5 (7–33) |
| Duration between disease confirmation and first semen analysis (days) | 77.2 ± 1.9 (75–81) |
| Duration between first and second semen analyses (days) | 92.8 ± 5.9 (81–110) |
| CO-RAD category by chest computed tomography |  |
| CO-RAD 2 | 25 (25%) |
| CO-RAD 3 | 54 (54%) |
| CO-RAD 4 | 21 (21%) |

CO-RAD: COVID-19 Reporting and Data System, COVID-19; Coronavirus disease 2019.
