# Supplementary material for: Semen quality changes during infection and recovery phases of mild-to-moderate COVID-19 in reproductive-aged patients: a prospective case series
Source: Basic Clin Androl. 2023 Jan 19;33:2. doi: 10.1186/s12610-022-00175-7 (PMC9848703; doi:10.1186/s12610-022-00175-7)
Supplement: Supplementary file 3 — Additional file 3. [file 12610_2022_175_MOESM3_ESM.docx]

**Table 2:** Comparison between semen parameters of the first (representing the infection phase) and second (representing the recovery phase) semen analyses^a^

| Semen parameters | First analysis | Second analysis | P-value |
| --- | --- | --- | --- |
|  | **Mean ± SD (Range) or Frequency (Percentage)** | |  |
| pH | 7.6 ± 0.3 (7–8) | 7.6 ± 0.3 (7–8) | 0.581 |
| Volume (mL) | 3.27 ± 0.75 (2–4.6) | 3.25 ± 0.76 (2–4.6) | 0.813 |
| Sperm concentration (million/mL) | 96.5 ± 35.9 (12–154) | 104.7 ± 33.8 (15–154) | 0.844 |
| Percentage of progressive sperm motility | 44.5 ± 6.7 (34–58) | 46.5 ± 7.1 (34–58) | 0.043 |
| Morphology (% of normal forms) | 23.4 ± 17.6 (1–55) | 30.6 ± 13 (2–60) | <0.001 |
| Red blood cells (10^6^/ml) | 1.07 ± 0.78 (0–2) | 1.06 ± 0.76 (0–2) | 0.877 |
| Leucocytes (10^6^/ml) | 1.2 ± 1 (0–6) | 1.1 ± 0.8 (0–2) | 0.539 |
| Total semen quality^b^ |  |  |  |
| Normal | 67 (67%) | 89 (89%) | <0.001 |
| Abnormal | 33 (33%) | 11 (11%) |  |

^a^ The pH and volume of semen and the progressive sperm motility were compared, using the paired Student-t test. The other parameters, including sperm concentration and morphology, red blood cells and leucocytes were compared by Wilcoxon test. In addition, the total semen quality was compared, using the Chi-squared test.

^b^ Total semen quality: This expression considered the quality of semen as a whole (normal or abnormal) relative to the presence or absence of the abnormality, regardless the number of the abnormal parameters.

Abbreviations: pH: the potential hydrogen describing the acidity or basicity of semen, SD: Standard deviation
